# Mega roles of microRNAs in regulation of skeletal muscle health and disease

**DOI:** 10.3389/fphys.2014.00239

**Published:** 2014-06-26

**Authors:** Mridula Sharma, Prasanna Kumar Juvvuna, Himani Kukreti, Craig McFarlane

**Affiliations:** ^1^Department of Biochemistry, YLL School of Medicine, National University of SingaporeSingapore, Singapore; ^2^Brenner Centre for Molecular Medicine, Singapore Institute for Clinical Sciences (A^*^STAR)Singapore, Singapore

**Keywords:** miRNAs, exercise, atrophy signaling, aging, myogenesis

## Abstract

Skeletal muscle is a dynamic tissue with remarkable plasticity. Skeletal muscle growth and regeneration are highly organized processes thus it is not surprising that a high degree of complexity exists in the regulation of these processes. Recent discovery of non-coding microRNAs (miRNAs) has prompted extensive research in understanding the roles of these molecules in skeletal muscle. Research so far shows that miRNAs play a very significant role at every aspect of muscle biology. Besides muscle growth, development, and regeneration miRNAs are also involved in the pathology of muscle diseases and metabolism. In this review, recent advancements in miRNA function during myogenesis, exercise, atrophy, aging, and dystrophy are discussed.

## MiRNA biogenesis

MiRNAs are short non-coding RNAs (~22 nucleotides), involved in the regulation of gene expression post-transcriptionally. Ever since the discovery of Lin-4 miRNA in *C. elegans*, by Lee et al. (1993), thousands of miRNAs and their targets have been identified. The advancements made in miRNA research prove that miRNAs are crucial for development, physiology and metabolism (Bushati and Cohen, 2007; Fineberg et al., 2009; Leung and Sharp, 2010). Distinct miRNAs potentially target an extensive set of mRNAs depending on their seed sequence and the target mRNA 3′ UTR sequence (Ambros, 2004; Bushati and Cohen, 2007; Bartel, 2009). MiRNAs are transcribed by Pol II or Pol III as individual miRNAs (monocistronic) or in clusters (polycistronic) from non-coding RNA genes (intergenic) or within the protein coding genes (intragenic). Most miRNAs are transcribed by RNA polymerase II as long primary miRNA (pri-miRNA) transcripts, which are then processed into ~70 nt (pre-miRNA) hairpin like RNA duplex by Drosha (Denli et al., 2004; Han et al., 2004, 2006). Pre-miRNAs are processed by Dicer in association with other RNA-binding proteins in cytoplasm giving rise to mature miRNA (Chendrimada et al., 2005; Lee et al., 2006). MiRNAs contribute to post-transcriptional regulation of gene expression by gene silencing. The seed sequence (2 to 8 nucleotides on the 5′ end of miRNA sequence) of miRNA base-pair to the target mRNAs within the 3′UTR or sometimes other mRNA regions in association with Argonaute proteins and normally lead to mRNA translational repression or destabilization and degradation (Meister and Tuschl, 2004; Du and Zamore, 2005; Zhao and Srivastava, 2007). Conserved seed sequence of miRNA enables targeting multiple mRNA targets; on the other hand a single mRNA can be targeted by more than one miRNA (Doench and Sharp, 2004; Brennecke et al., 2005; Lewis et al., 2005; Grimson et al., 2007; Bartel, 2009; Brodersen and Voinnet, 2009).

Several miRNAs are ubiquitously expressed where as some are tissue specific and some are enriched in specific tissues. MiRNAs miR-1, miR-133a, miR-133b, miR-206, miR-208, miR-208b, miR-486, and miR-499 are Muscle enriched miRNAs so called myomiRs (McCarthy and Esser, 2007; Callis et al., 2008). Muscle regulatory factors (MRFs) like Myf5, Myogenin, MyoD, and Mrf4 play a very significant role in myogenesis. MRFs are involved in regulation of myomiR expression (Zhao et al., 2005; Rao et al., 2006). These myomiRs are differentially regulated during myogenesis, development, degeneration, atrophy, various myopathies, and exercise. In addition to the myomiRs, other miRNAs are implicated in myogenesis and muscle pathology (Table 1); hence in this review functions of both myomiRs and other miRNAs in skeletal muscle are discussed.

**Table 1:** **Over view of miRNAs differentially regulated in myogenesis, exercise, atrophy, aging, and Duchenne muscular dystrophy and their functional roles in regulation, maintenance, and pathology of skeletal muscle**.

**Condition**	**MicroRNA**	**Function**
Myogenesis	miR-1	Promotes myoblast differentiation and regeneration by down regulating HDAC4 that represses MEF2- activated muscle gene expression.
	miR-23a	Inhibits myogenic differentiation by silencing Myosin heavy chain 1, 2, and 4 expression.
	miR-27a/b	Induce skeletal muscle hypertrophy by down regulating Myostatin, an inhibitor of myogenesis.
	miR-29	Enhances muscle differentiation by repressing polycomb proteins. MiR-29 also inhibits proliferation by down regulating Akt3 *in-vitro.*
	miR-133	Enhances myoblast proliferation by negatively regulating SRF.
	miR-206	Promotes myoblast differentiation and regeneration by targeting Pax7.
	miR-675-3p and miR-675-5p	Induce myogenesis and differentiation of satellite cells during regeneration.
Exercise		miRNAs in skeletal muscle and serum are differentially regulated depending on type of exercise, age of the subjects and the duration of exercise. However, myomiRs miR-1, miR-133, miR-208, and miR-486 are upregulated in most of the exercise regimens indicating their role in hypertrophy, regeneration, and maintenance of healthy skeletal muscle.
Atrophy	MyomiRs	Myomirs, miR-1-1, miR-1-2, miR-133a, miR-133b, and miR-206 are downregulated in TWEAK induced muscle wasting.
	miR-1	Upregulated in Dex induced muscle atrophy and targets HSP70 leading to upregulation of MuRF1 and Atrogin-1.
	miR-23a	Confers resistance to skeletal muscle atrophy by post-transcriptional regulation of Atrogin1 and MuRF1.
	miR-29	Reduced levels in muscle of mice with Chronic kidney diseases (CKD) lead to increased levels of YY1 protein inhibiting the satellite cell differentiation and muscle regeneration.
	miR-146a	Elevated expression during muscle wasting leads to downregulation of Numb and TRAF6 involved in satellite cell activation and AKT signaling respectively resulting in impaired regeneration.
	miR-486	Mimics for miR-486 rescued muscle atrophy in CKD mice by negatively regulating FoxO1 and PTEN.
Aging	miR-29	Upregulated during aging, inhibits muscle regeneration by targeting IGF-1 and p85α that decreases the overall protein translation during aging.
	miR-181	Decreased levels during aging lead to higher levels of targets like TNF-α, IL-6, IL-1β, and IL-8 in the muscle during aging.
	miR-206	Increased levels in aging skeletal muscle could be conferring resistance to muscle atrophy.
Duchenne muscular dystrophy	miR-29	Reduced levels of miR-29 result in decreased muscle regeneration and increased fibrogenesis in mdx muscle.
	miR-31	Higher levels of miR-31 augment pathology of dystrophic muscle.
	miR-145 and -133a	Increased serum levels are potential biomarkers in a mouse model of DMD.
	miR-199a-5p	Increased levels in dystrophic muscle affect myogenesis by targeting WNT signaling proteins necessary for cell proliferation and differentiation.
	miR-206	Elevated levels in serum are a potential biomarker for DMD.
	miR-486	Reduced levels in mdx mice affect cell cycle and muscle regeneration by targeting PTEN/AKT pathway and platelet-derived growth factor receptor β.

Functional significance of miRNAs in myogenesis, exercise, disease, and aging of skeletal muscle.

## MiRNAs in myogenesis

A cluster of myomiRs including miR-1, miR-133, and miR-206 plays a role in myogenesis, and muscle regeneration (Rao et al., 2006). Myogenesis involves proliferation and differentiation of myoblasts during pre- and postnatal stages. MiR-1 represses histone deacetylase 4 (HDAC4), which is a transcriptional repressor of MEF2- activated muscle gene expression, hence promotes myoblast differentiation whereas miR-133 enhances myoblast proliferation by targeting serum response factor (SRF), a transcription factor involved in muscle differentiation (Miano, 2003; Jian-Fu et al., 2005). On the other hand, miR-206 promoted differentiation of myoblasts *in-vitro* by down-regulation of Pola 1, a subunit of DNA polymerase α (Kim et al., 2006). DNA polymerase α is necessary for cell proliferation and thus inhibition of Pola 1 by miR-206 resulted in inhibition of myoblast cell cycle. Additionally, miR-206 might indirectly down regulate DNA-Binding Protein Inhibitor (Id1-3) and Myogenic Repressor (MyoR), inhibitors of myogenic transcription factors like MyoD and regulate myoblast differentiation (Kim et al., 2006). The promyogenic activities of miR-206 and -486 were also evident in a study by Dey et al. (2011), in which they showed that miR-206 and -486 promoted myoblast differentiation by targeting Pax7, a paired box transcription factor that inhibits differentiation by negatively regulating MyoD levels. Moreover MyoD was shown to induce the expression of miR-206 and -486 (Dey et al., 2011).

MiR-23a, a ubiquitous miRNA was shown to inhibit myogenic differentiation by targeting the 3′ UTRs of fast Myosin heavy chain 1, 2, and 4 transcripts (Wang et al., 2012a,b). Many studies have suggested that miR-29 is a positive regulator of myogenesis. Huating et al. (2008) deciphered that NF-kB repressed miR-29 through Yin Yang 1 (YY1) protein, a part of Polycomb complex during myoblast proliferation. During myogenesis, when NF-kB and subsequently YY1 levels were reduced, miR-29 expression was stimulated. MiR-29 was then able to enhance differentiation by inhibiting YY1 in the C2C12 myoblasts. Furthermore, miR-29 expression was downregulated in Rhabdomyosarcomas (RMS) through epigenetic silencing and its overexpression was able to rescue differentiation in RMS cell line (Huating et al., 2008). Another component of Polycomb repressive complex, RING1, and YY1 binding protein (Rybp) has the target sequence for miR-29 in its 3′ UTR. Moreover, down-regulation of Rybp in C2C12 and regenerating skeletal muscle coincided with myogenesis indicating that Rybp inhibits C2C12 myoblast differentiation and muscle regeneration. The Rybp and YY1 complex was found to be present on many myogenic gene promoters including miR-29 suggesting the presence of Rybp-miR-29 feedback loop. Concomitant with increased expression of Rybp, an accumulation of Enhancer of zeste homolog 2 (Ezh2) and trimethylation of H3K27 at myogenic gene loci has been observed (Zhou et al., 2012). These findings clearly show that miR-29 mediates the repression of Polycomb complex consequently influencing the chromatin of myogenic genes during skeletal muscle differentiation. In a recent study, Wei et al. (2013) showed that miR-29 inhibited myoblast proliferation and induced differentiation through a more direct mechanism by down regulating Akt3 (a member of the serine/threonine protein kinase family responsive to growth factor cell signaling). Overexpression of Akt3 increased myoblast proliferation and reduced myoblast differentiation and resisted miR-29 mediated inhibition (Wei et al., 2013).

Skeletal muscle cell differentiation was also inhibited when miR-186 was overexpressed in myoblasts. MiR-186 was shown to directly target myogenin, which is a key regulator of skeletal muscle differentiation (Antoniou et al., 2014). Recently, an interesting mechanism involved in myoblast differentiation has been reported by de la Garza-Rodea et al. (2014) whereby S1P lyase (SPL) an enzyme that degrades sphingosine-1-phosphate (S1P) was shown to regulate myoblast differentiation by regulating the expression of miR-1, miR-206, and miR-486 (de la Garza-Rodea et al., 2014). Another novel discovery is of miR-675-3p and miR-675-5p, which are coded in the exon1 of long non-coding RNA H19, were shown to induce myogenesis and differentiation of satellite cells during regeneration. MiR-675-3p and miR-675-5p target the 3′UTRs of Smads and DNA replication initiation factor Cdc6 thus allowing the myoblasts to differentiate and regenerate the muscle (Dey et al., 2014).

One other miRNA that has been observed to play a role in myogenesis is miR-27a/b. MiR-27a/b negatively regulates Myostatin, an inhibitor of myogenesis (Huang et al., 2012; McFarlane et al., 2014). Antagonization of miR-27a/b led to higher Myostatin expression, decreased myoblast proliferation, and reduced satellite cell activation. While overexpression of miR-27a/b downregulated Myostatin and induced skeletal muscle hypertrophy (McFarlane et al., 2014).

Skeletal muscle is also known for its remarkable ability to undergo muscle regeneration by virtue of satellite cells. Satellite cells stay quiescent in resting muscle however once stimulated; enter cell cycle and differentiation program. Satellite cells are known to express quiescence marker genes such as Pax7. Consistently, miR-1 and miR-206 were shown to be upregulated in satellite cells after muscle injury and induced muscle regeneration by targeting Pax7 (Chen et al., 2010). Recently, miR-31 was shown to maintain satellite cells in quiescent state through the regulation of Myf5 mRNA in mRNP granules. Once the satellite cells were activated miR-31was degraded in the mRNP granules, Myf5 mRNA became available for translation and Myf5 protein levels increased in the cells to initiate differentiation (Crist et al., 2012). This remarkable mechanism keeps the satellite cells in quiescent but “ready” state to enter cell cycle for muscle growth and regeneration.

## MiRNAs and exercise

Skeletal muscle is a highly contractile tissue and physical activity is essential for maintaining muscle mass. It is apparent that exercise is a potent stimulus for satellite cell activation and muscle protein synthesis. Specifically, resistance exercise increases skeletal muscle mass by hypertrophy of muscle fibers (Mayhew et al., 2009; Stepto et al., 2009; Dreyer et al., 2010). Previous studies have indicated that exercise induces changes both at the genetic and epigenetic levels. Several miRNAs are differentially regulated during and after exercise, and some miRNAs are secreted in to circulation (Baggish et al., 2011; Davidsen et al., 2011).

The expression of myomiRs miR-1 and miR-133a was upregulated in young men soon after an acute exposure to endurance exercise (Nielsen et al., 2010). The increased expression of these myomiRs after acute endurance exercise correlates with upregulated levels of MyoD, myogenin, and MRF4, the myogenic transcription factors involved in myomiR expression (Kadi et al., 2004; Sweetman et al., 2008). It is noteworthy that within a fortnight of discontinuation of exercise, the myomiR levels were similar to the pre-exercise levels. However, the downstream targets and the signaling mechanisms affected by the myomiRs in this exercise regimen are yet to be confirmed. In an attempt to understand the basis of variable response of individuals to resistance exercise, differential expression of 21 abundant miRs was determined in the muscle of men subjected to resistance training for 12 weeks (Davidsen et al., 2011). After the training low responders did not gain any noticeable muscle mass and miR-378, miR-29a, and miR-26a levels were reduced while miR-451 was higher in the muscle of low responders (Davidsen et al., 2011). Interestingly, the expression of miR-378, miR-29a, and miR-26a was unaltered in the muscle of high responders which was alluded to be a compensatory effect. The results of this study show a correlation between the levels of miR-378 and muscle hypertrophy induced by exercise. Russell et al. (2013) reported that miR-1, miR-133a/b, and miR-181a were higher and miR-9, miR-23a, miR-23b, and miR-31 were lower in the muscle of subjects after 3 h of an acute level of endurance cycling (Russell et al., 2013). The mRNA levels of Drosha, Dicer and Exportin involved in miRNA biogenesis were also up regulated.

It appears that exercise not only affects miRNA expression in the muscle tissue but also in circulation. After acute resistance exercise, miR-149^*^ expression was more while miR-146a and miR-221 expression was lower in circulation of the subjects (Sawada et al., 2013). It might be that decrease in miR-146a and miR-221 allows the muscle regeneration to proceed since these miRNAs affect differentiation (Cardinali et al., 2009; Kuang et al., 2009). Aoi et al. (2013) observed that circulatory levels of miR-486 were reduced after acute and chronic exercise in young men. MiR-486 is expressed at a higher level in muscle than other tissues and was shown to target Phosphatase and tensin homolog (PTEN), an inhibitor of PI3K/AKT pathway (Small et al., 2010). The PI3K/AKT pathway is downstream of Insulin signaling therefore miR-486 can regulate glucose uptake through PTEN in skeletal muscle. Based on these results, Aoi et al. (2013) proposed that miR-486 could be playing a role in exercise induced metabolic adaptations (Aoi et al., 2013). Alexander et al. (2013a,b) analyzed the expression profile of miRNAs and their regulatory targets in athletes who performed 30 min of exercise followed by a rest for 30 or 60 min. It is noteworthy that circulatory miR-181a-5p and clustered miRs miR-27a-5p and miR-24-2-5p were up regulated immediately after exercise followed by a decrease during the relaxation. The expression of some of the target genes of these miRNAs indicated that these miRNAs might be playing a role in adaptation during exercise (Alexander et al., 2013a).

MyomiRs miR-1, miR-133a and cardiac miR-208a were robustly upregulated in the circulation of subjects soon after aerobic exercise like marathon and regressed to normal levels 24 h after the marathon. On the other hand, the proteins involved in skeletal muscle injury, cardiac damage, and inflammation were upregulated after marathon and continued to be higher 24 h after marathon. These results suggest that circulatory miRNAs may be indicators of real-time changes induced by exercise and oxidative ability of muscles (Baggish et al., 2014; Mooren et al., 2014). Recently, Nielsen et al., 2014 compared the expression of miRNAs after acute and endurance exercise and discovered a pattern of miRNAs, which was unique to each type of exercise (Nielsen et al., 2014).

## MiRNAs in muscle atrophy

Muscle atrophy is characterized by loss of muscle mass. The loss of muscle mass is due to either enhanced degradation of muscle proteins and or reduced protein synthesis in skeletal muscle. Enhanced degradation of muscle sarcomeric proteins, myosin heavy chains (MyHC) is mediated through E3 ubiquitin ligases Atrogin-1 and MuRF1 (Lecker et al., 2004; Sacheck et al., 2007; Lokireddy et al., 2012; Sriram et al., 2014). Several muscle atrophy-inducing stimuli have been reported to stimulate these ubiquitin ligases. Recently, post-transcriptional regulation of Atrogin-1 and MuRF1 through miR-23a has been observed (Wada et al., 2011) indicating that miR-23a is a regulator of muscle atrophy. Indeed, overexpression of miR-23a in myotubes and skeletal muscle resulted in resistance to glucocorticoid Dexamethasone (Dex) induced muscle atrophy (Wada et al., 2011). MiR-23a was also found to be downregulated due to reduced calcineurin/NFAT signaling in the muscle of Dex treated mice (Hudson et al., 2014). Dex treatment was also found to enhance its exosomal packaging.

High doses of Dex or Myostatin induce severe skeletal muscle atrophy. In our recent publication we described the role of miR-1 in Dex-mediated atrophy (Kukreti et al., 2013). In this study we have shown a novel miR-1-mediated mechanism through which Dex promotes skeletal muscle atrophy by targeting the protective protein HSP70. We showed glucocorticoid receptor mediated miR-1 upregulation after both Dex and Myostatin treatment in C2C12 myotubes and animal models of Dex-induced muscle atrophy. Inhibition of miR-1 in C2C12 myotubes attenuated the Dex-induced increase in atrophy related proteins confirming a role for miR-1 in atrophy. Increased miR-1 during atrophy reduced HSP70 levels, which resulted in decreased phosphorylation of AKT, due to loss of HSP70 bound pAKT. The loss of pAKT lead to decreased phosphorylation, and thus, enhanced activation of Forkhead box O3 (FoxO3) and upregulation of MuRF1 and Atrogin-1. Thus, we proposed a model whereby Dex and Myostatin mediated atrophic signals were integrated through miR-1, which then directly or indirectly, inhibited the proteins involved in resisting atrophy.

Chronic kidney diseases (CKD) are associated with muscle wasting. Besides the mechanisms of protein degradation and reduced protein synthesis, reduced muscle satellite cell (stem cell) function also plays a role in muscle atrophy. Several genetic and epigenetic processes that affect satellite cell function have been recognized previously. Not so long ago, Wang et al. (2012a,b) demonstrated that satellite cell function is impaired in the muscles of CKD mice. The differentiation of these satellite cells was impaired due to an increase in YY1 protein, a well-established inhibitor of myogenesis. YY1 protein was found to be upregulated in the satellite cells of CKD muscle. Microarray analysis of the muscle from mice with CKD revealed that miR-29 expression was downregulated in the satellite cells. One of the targets of miR-29 is YY1; hence reduction in miR-29 expression released the repression on YY1 and caused poor muscle differentiation and regeneration. Overexpression of miR-29 in the C2C12 cells enhanced the differentiation and rescued the repression in YY1 overexpressing cells indicating miR-29-mediated regulation of YY1 and myoblast differentiation.

To circumvent the CKD induced atrophy, Jing et al. (2012) used miR-486 mimic and transfected in the muscle of mice with CKD. They observed that miR-486 mimic was able to rescue the atrophy phenotype despite CKD. Two important targets of miR-486 are Forkhead box O1 (FoxO1) and PTEN. PTEN is known to dephosphorylate AKT and dephosphorylation of AKT leads to dephosphorylation of FoxO1 and thus its nuclear translocation. Furthermore, miR-486 inhibits FoxO1 at the protein level. These two mechanisms combined together effectively render the FoxO1 mediated atrophy signaling inactive in miR-486 mimic treated CKD affected muscle and Dex treated myotubes (Jing et al., 2012).

Several cytokines play a role in inducing atrophy in skeletal muscle. Newly found cytokine TNF-like weak inducer of apoptosis (TWEAK) belongs to TNF family and has been shown to cause muscle wasting (Panguluri et al., 2010). One of the mechanisms proposed for induction of muscle wasting by TWEAK is via regulation of differential expression of several miRNAs in myotubes. Myomirs including miR-1-1, miR-1-2, miR-133a, miR-133b, and miR-206 were downregulated while miR-146a and miR-455 were upregulated upon treatment of myotubes with TWEAK protein. Panguluri et al. (2010) suggested that reduced levels of Myomirs would affect myogenesis during muscle wasting and increased levels of miR-146a would target genes like Numb and TRAF6. Numb is involved in satellite cell activation and downregulation of numb by miR-146a would negatively affect muscle regeneration. TRAF6 has been shown to activate AKT signaling that is known to enhance protein synthesis and thus hypertrophy signaling.

Muscle atrophy is also associated with fiber type changes consistent with slow to fast fiber type switching (Baldwin and Haddad, 2002). The fiber type change is manifested through the downregulation of slow twitch beta-myosin heavy chain (β-MyHC) and an increase in fast twitch MyHC (Type IIb and IIx) (Stevenson et al., 2003). Earlier, transcription factors, Purα, Pur β, and SP3, which regulate β-MyHC expression during atrophy, were identified. Recently miR circuitry regulating β-MyHC at the post-transcriptional level has been discovered. Introns of Myh6 (α-MHC), Myh7, and Myh7b encode miR-208a, -208b, and -499 respectively and these miRs have been shown to regulate MyHC levels. Mir-208a has been shown to target a thyroid hormone receptor cofactor (Thrap1), which is known to repress β-MyHC levels indicating that miR-208a activates β-MyHC gene expression indirectly (Van Rooij et al., 2008). The same group has also reported that miR-499 was instrumental in inducing fast-to-slow muscle fiber switching phenotype by reducing the expression of Sox6 transcription factor. Sox6, a repressor of β-MyHC gene expression increased during atrophy. Additionally, miR-208a and b can regulate the expression of miR-499 through its host gene β-MyHC gene expression by repressing the transcription factor, e.g., Sox6. These miRNAs are an excellent example of circuitry (McCarthy et al., 2009).

In another study, levels of miR-696 were up regulated in the muscles of mice subjected to hind limb immobilization while levels of PGC-1alpha, a target of miR-696 were decreased. Consistent with this observation miR-696 over expressing myocytes showed a decrease in PGC-1alpha, which is involved in mitochondrial function and metabolism (Liang and Ward, 2006). Furthermore, the expression of miR-696 was reduced in the muscles of mice after exercise and the levels of PGC-1 alpha were increased. These results indicate the importance of miR-696 in skeletal muscle atrophy, metabolism and adaptations (Aoi et al., 2010).

## MiRNAs in skeletal muscle aging

Naturally occurring muscle wasting during aging is known as sarcopenia. Sarcopenia starts from the age of forty years in humans and gets worse with each decade. Even though it is known to exist, the mechanisms behind sarcopenia are not fully known. With increasing understanding of miRs, it is apparent that miRs also a play a role during aging.

Drummond et al. (2008) analyzed the expression of myomirs, miR-1, miR-133, and miR-206 in the skeletal muscle of old and young humans subjected to exercise and administration of leucine rich amino acid solution. The results indicated that basal expression of pri-miRNA-1-1, -1-2, -133a-1, and -133a-2 was higher in older compared with young men. Furthermore, these primary miRNAs were downregulated at 6 h after exercise only in the young men. Finally, it was inferred that an increased primary miRNA expression occurs during aging; the effect of anabolic stimulus on miRNA levels was perturbed in older men. Interestingly, the expression of primary miR-206 continued to be higher in older men even after anabolic stimulus however the expression of mature miR-206 did not alter (Drummond et al., 2008).

Subsequently, Hamrick et al. (2010) carried out a microarray analysis in the muscles of 12 and 24 month old mice and observed that miR-206, -698, and -468 increased during aging and miR-434, -455, -382, -181a, and -221 were reduced (Hamrick et al., 2010). Clop et al have demonstrated the role of miR-206 in skeletal muscle hypertrophy previously (Clop et al., 2006), therefore the assumption for the action of miR-206 during aging is to protect muscle from atrophy. The targets for miR-698 and -468 have not been validated in skeletal muscle yet; Hamrick et al. predicted that cardiotrophin1 could be the target for miR-698 in skeletal muscle since cardiotrophin1 was found to inhibit myogenic regulatory factors during differentiation (Miyake et al., 2009). In line with the protective roles of some of the miRs in skeletal muscle during aging, miR-221 also fits in that role. Higher levels of miR-221 inhibit differentiation of myoblasts and during normal course of myoblast differentiation its expression is reduced. Taken together, these miRNAs are helping skeletal muscle to maintain the differentiation phenotype during aging. Mir-455 has been shown to be involved in brown adipocyte differentiation (Walden et al., 2009) and how its downregulation in aging skeletal muscle would affect the muscle function is a matter of speculation.

A recent RNA sequencing study by Mercken et al. (2013) revealed differential expression of miRs in the skeletal muscles of old and young rhesus monkeys. They observed an increase in the expression of several novel miRNAs including miR-744-5p in the old skeletal muscle. The expression of myomir, miR-181a was downregulated and calorie restriction was able to revert the levels of miR-181a in the old muscle. Based on the earlier reports showing Tumor Necrosis Factor-alpha (TNF-α), Interleukin-6 (IL-6), Interleukin-1beta (IL-1β), and Interleukin-8 (IL-8) as the targets of miR-181a Hutchison et al. (2013); Xie et al. (2013) and Mercken et al. (2013) proposed that decrease in miR-181a could be responsible for an increase in the mentioned inflammatory cytokines in the muscle during aging.

Similarly, microarray analysis of miRNAs in muscles from young (4 months) and old (28 months) rats was shown to alter (Hu et al., 2014a,b). Several miRNAs were up or down regulated. MiR-29 has been seen to be consistently upregulated during sarcopenia, senescence, or aging. In the aged rat muscle, miR-29 was shown to target Insulin like Growth Factor-1 (IGF-1), Phosphatidylinositide 3-kinase p85 alpha regulatory subunit (p85α) and Myeloblastosis-related protein B (B-myb) transcripts. By targeting IGF-1 and p85α proteins, miR-29 inhibited muscle protein synthesis and myogenesis since IGF-1 and p85α are well established signaling molecules for protein translation and a decrease in these proteins was observed during muscle atrophy including sarcopenia (Owino et al., 2001; Barbour et al., 2005; Park et al., 2009; Smith et al., 2012). Overexpression of miR-29 in skeletal muscle also resulted in an increase in cell cycle arrest proteins, Cyclin-Dependent kinase inhibitor 2A, (p16Ink4A) and Retinoblastoma protein (pRB) in agreement with the increased expression of these proteins in aged muscle (Hu et al., 2014a,b). Thus, miR-29 regulates the levels of various proteins directly or indirectly eventually resulting in muscle atrophy during aging.

## MiRNAs in duchenne muscular dystrophy

Duchenne muscular dystrophy (DMD) is caused by mutations in dystrophin gene leading to progressive muscle wasting. In DMD pathology muscle tissue undergoes severe cellular and molecular changes. Several studies have determined the expression of myomiRs, miR-1, -133, and -206 levels in serum of DMD patients and found that these myomiRs were upregulated. Consistently, miR-206 correlates with DMD pathology and thus could be a useful biomarker for DMD along with creatine kinase (Cacchiarelli et al., 2011; Mizuno et al., 2011; Vignier et al., 2013; Zaharieva et al., 2013; Hu et al., 2014a,b; Roberts et al., 2014). Furthermore, miR-145 and miR-133a were shown to be potential biomarkers in a mouse model of DMD (Endo et al., 2013).

Microarray analyses of miRNAs have been conducted in the muscles from DMD patients and dystrophic animal models. There have been some inconsistencies in the results due to the type of muscle used and animal model itself. Intriguingly, the miRNAs expressed in diaphragm of X chromosome-linked muscular dystrophy (mdx) mice were not significantly different from the wild type diaphragm (Greco et al., 2009; Thomas et al., 2012). MyomiR-486 expression was reduced in mdx muscle. MiR-486 was shown to regulate PTEN/AKT pathway and platelet-derived growth factor receptor β thus affecting the cell cycle and muscle regeneration in mdx muscle (Alexander et al., 2011). Mdx mice also showed reduced levels of miR-29 (Eisenberg et al., 2007; Greco et al., 2009). MiR-29 has been shown to positively regulate myogenesis and reduce fibrogenesis (Lijun et al., 2012). Indeed, overexpression of miR-29 in mdx muscle increased regeneration concomitant with decreased fibrosis (Lijun et al., 2012). Increased expression of miR-31 was reported in DMD patients and mdx mice (Greco et al., 2009; Thomas et al., 2012; Roberts et al., 2014). MiR-31 has been shown to regulate the dystrophin levels by targeting the 3' UTR of dystrophin. Hence, Cacchiarelli et al. (2011) have proposed that inhibition of miR-31 would improve dystrophin expression during treatment of dystrophy through exon skipping. MiR-199a-5p levels were increased in human dystrophic skeletal muscle. MiR-199a-5p regulates the proteins of WNT signaling pathway namely Frizzled 4 (FZD4), Jagged1 (JAG1), and WNT2. These proteins play a role in cell proliferation and differentiation thus downregulation of these proteins by miR-199a-5p would affect myogenesis in dystrophic muscle (Alexander et al., 2013b).

## Perspective

It is evident that miRNAs are a critical component of regulatory mechanisms in muscle (Table 1). MiRNAs also appear to be versatile in their function for example miR-1 has a role in myogenesis as well as muscle atrophy. These findings also suggest that miRNAs could have context specific function. It is fascinating to note that miRNAs are not simply regulating their targets post-transcriptionally but also in many instances their own transcription via various circuitries. Many miRNAs are involved in disease conditions therefore strategies for usage of miRNAs or antagonists could be developed for therapeutic purposes. Indeed, mimics for miR-34a are being tried in a Phase I trial by Mirna Therapeutics to increase expression of miR-34a in tumors and Miravirsen (Santaris Pharma), a LNA-modified oligonucleotide inhibitor of miR-122 has been tried in humans for the treatment of Hepatitis-C (Hydbring and Badalian-Very, 2013). As per these results, utility of miRNA-based therapeutics appears to be encouraging. Furthermore, the fact that expression of miRNAs can be regulated by external cues such as exercise, incorporation of such external cues in therapeutic approaches needs to be considered.

### Conflict of interest statement

The authors declare that the research was conducted in the absence of any commercial or financial relationships that could be construed as a potential conflict of interest.

## References

[B1] Alexander G. T. Diana V. M. Asghar A. Timur R. S. Dmitry A. S. Maxim U. S. (2013a). Dynamically regulated miRNA-mRNA networks revealed by exercise. BMC Physiol. 13:9 10.1186/1472-6793-13-9 24219008 PMC3681679

[B2] Alexander M. S. Casar J. C. Motohashi N. Myers J. A. Eisenberg I. Gonzalez R. T. (2011). Regulation of DMD pathology by an ankyrin-encoded miRNA. Skelet. Muscle 1:27 10.1186/2044-5040-1-27 21824387 PMC3188430

[B3] Alexander M. S. Kawahara G. Motohashi N. Casar J. C. Eisenberg I. Myers J. A. (2013b). MicroRNA-199a is induced in dystrophic muscle and affects WNT signaling, cell proliferation, and myogenic differentiation. Cell Death Differ. 20, 1194–1208 10.1038/cdd.2013.62 23764775 PMC3741500

[B4] Ambros V. (2004). The functions of animal microRNAs. Nature 431, 350–355 10.1038/nature02871 15372042

[B5] Antoniou A. Mastroyiannopoulos N. P. Uney J. B. Phylactou L. A. (2014). miR-186 inhibits muscle cell differentiation through myogenin regulation. J. Biol. Chem. 289, 3923–3935 10.1074/jbc.M113.507343 24385428 PMC3924261

[B6] Aoi W. Ichikawa H. Mune K. Tanimura Y. Mizushima K. Naito Y. (2013). Muscle-enriched microRNA miR-486 decreases in circulation in response to exercise in young men. Front. Physiol. 4:80 10.3389/fphys.2013.00080 23596423 PMC3622901

[B7] Aoi W. Naito Y. Mizushima K. Takanami Y. Kawai Y. Ichikawa H. (2010). The microRNA miR-696 regulates PGC1-alpha in mouse skeletal muscle in response to physical activity. Am. J. Physiol. Endocrinol. Metab. 298, E799–E806 10.1152/ajpendo.00448.2009 20086200

[B8] Baggish A. L. Hale A. Weiner R. B. Lewis G. D. Systrom D. Wang F. (2011). Dynamic regulation of circulating microRNA during acute exhaustive exercise and sustained aerobic exercise training. J. Physiol. 589, 3983–3994 10.1113/jphysiol.2011.213363 21690193 PMC3179997

[B9] Baggish A. L. Park J. Min P.-K. Isaacs S. Parker B. A. Thompson P. D. (2014). Rapid upregulation and clearance of distinct circulating microRNAs after prolonged aerobic exercise. J. Appl. Physiol. 116, 522–531 10.1152/japplphysiol.01141.2013 24436293 PMC3949215

[B10] Baldwin K. M. Haddad F. (2002). Skeletal muscle plasticity: cellular and molecular responses to altered physical activity paradigms. Am. J. Phys. Med. Rehabil. 81, S40–S51 10.1097/00002060-200211001-00006 12409810

[B11] Barbour L. A. Mizanoor Rahman S. Gurevich I. Leitner J. W. Fischer S. J. Roper M. D. (2005). Increased P85α is a potent negative regulator of skeletal muscle insulin signaling and induces *in vivo* insulin resistance associated with growth hormone excess. J. Biol. Chem. 280, 37489–37494 10.1074/jbc.M506967200 16166093

[B12] Bartel D. P. (2009). MicroRNAs: target recognition and regulatory functions. Cell 136, 215–233 10.1016/j.cell.2009.01.002 19167326 PMC3794896

[B13] Brennecke J. Stark A. Russell R. B. Cohen S. M. (2005). Principles of microRNA-target recognition. PLoS Biol. 3:e85 10.1371/journal.pbio.0030085 15723116 PMC1043860

[B14] Brodersen P. Voinnet O. (2009). Revisiting the principles of microRNA target recognition and mode of action. Nat. Rev. Mol. Cell Biol. 10, 141–148 10.1038/nrm2619 19145236

[B15] Bushati N. Cohen S. M. (2007). microRNA functions. Annu. Rev. Cell Dev. Biol. 23, 175–205 10.1146/annurev.cellbio.23.090506.123406 17506695

[B16] Cacchiarelli D. Legnini I. Martone J. Cazzella V. Amico A. Bertini E. (2011). miRNAs as serum biomarkers for Duchenne muscular dystrophy. EMBO Mol. Med. 3, 258–265 10.1002/emmm.201100133 21425469 PMC3112257

[B17] Callis T. E. Deng Z. Chen J.-F. Wang D.-Z. (2008). Muscling through the microRNA world. Exp. Biol. Med. 233, 131–138 10.3181/0709-mr-237 18222968

[B18] Cardinali B. Castellani L. Fasanaro P. Basso A. Alemà S. Martelli F. (2009). Microrna-221 and microrna-222 modulate differentiation and maturation of skeletal muscle cells. PLoS ONE 4:e7607 10.1371/journal.pone.0007607 19859555 PMC2762614

[B19] Chen J.-F. Tao Y. Li J. Deng Z. Yan Z. Xiao X. (2010). microRNA-1 and microRNA-206 regulate skeletal muscle satellite cell proliferation and differentiation by repressing Pax7. J. Cell Biol. 190, 867–879 10.1083/jcb.200911036 20819939 PMC2935565

[B20] Chendrimada T. P. Gregory R. I. Kumaraswamy E. Norman J. Cooch N. Nishikura K. (2005). TRBP recruits the Dicer complex to Ago2 for microRNA processing and gene silencing. Nature 436, 740–744 10.1038/nature03868 15973356 PMC2944926

[B21] Clop A. Marcq F. Takeda H. Pirottin D. Tordoir X. Bibe B. (2006). A mutation creating a potential illegitimate microRNA target site in the myostatin gene affects muscularity in sheep. Nat. Genet. 38, 813–818 10.1038/ng1810 16751773

[B22] Crist C. G. Montarras D. Buckingham M. (2012). Muscle satellite cells are primed for myogenesis but maintain quiescence with sequestration of Myf5 mRNA targeted by microRNA-31 in mRNP Granules. Cell Stem Cell 11, 118–126 10.1016/j.stem.2012.03.011 22770245

[B23] Davidsen P. K. Gallagher I. J. Hartman J. W. Tarnopolsky M. A. Dela F. Helge J. W. (2011). High responders to resistance exercise training demonstrate differential regulation of skeletal muscle microRNA expression. J. Appl. Physiol. 110, 309–317 10.1152/japplphysiol.00901.2010 21030674

[B24] de la Garza-Rodea A. S. Baldwin D. M. Oskouian B. Place R. F. Bandhuvula P. Kumar A. (2014). Sphingosine phosphate lyase regulates myogenic differentiation via S1P receptor-mediated effects on myogenic microRNA expression. FASEB J. 28, 506–519 10.1096/fj.13-233155 24158395 PMC3868843

[B25] Denli A. M. Tops B. B. Plasterk R. H. Ketting R. F. Hannon G. J. (2004). Processing of primary microRNAs by the Microprocessor complex. Nature 432, 231–235 10.1038/nature03049 15531879

[B26] Dey B. K. Gagan J. Dutta A. (2011). miR-206 and -486 induce myoblast differentiation by downregulating Pax7. Mol. Cell. Biol. 31, 203–214 10.1128/mcb.01009-10 21041476 PMC3019853

[B27] Dey B. K. Pfeifer K. Dutta A. (2014). The H19 long noncoding RNA gives rise to microRNAs miR-675-3p and miR-675-5p to promote skeletal muscle differentiation and regeneration. Genes Dev. 28, 491–501 10.1101/gad.234419.113 24532688 PMC3950346

[B28] Doench J. G. Sharp P. A. (2004). Specificity of microRNA target selection in translational repression. Genes Dev. 18, 504–511 10.1101/gad.1184404 15014042 PMC374233

[B29] Dreyer H. C. Fujita S. Glynn E. L. Drummond M. J. Volpi E. Rasmussen B. B. (2010). Resistance exercise increases leg muscle protein synthesis and mTOR signaling independent of sex. Acta Physiol. 199, 71–81 10.1111/j.1748-1716.2010.02074.x 20070283 PMC2881180

[B30] Drummond M. J. McCarthy J. J. Fry C. S. Esser K. A. Rasmussen B. B. (2008). Aging differentially affects human skeletal muscle microRNA expression at rest and after an anabolic stimulus of resistance exercise and essential amino acids. Am. J. Physiol.Endocrinol. Metab. 295, E1333–E1340 10.1152/ajpendo.90562.2008 18827171 PMC2603551

[B31] Du T. Zamore P. D. (2005). microPrimer: the biogenesis and function of microRNA. Development 132, 4645–4652 10.1242/dev.02070 16224044

[B32] Eisenberg I. Eran A. Nishino I. Moggio M. Lamperti C. Amato A. A. (2007). Distinctive patterns of microRNA expression in primary muscular disorders. Proc. Natl. Acad. Sci. U.S.A. 104, 17016–17021 10.1073/pnas.0708115104 17942673 PMC2040449

[B33] Endo K. Weng H. Naito Y. Sasaoka T. Takahashi A. Fukushima Y. (2013). Classification of various muscular tissues using miRNA profiling. Biomed. Res. 34, 289–299 10.2220/biomedres.34.289 24389405

[B34] Fineberg S. K. Kosik K. S. Davidson B. L. (2009). MicroRNAs potentiate neural development. Neuron 64, 303–309 10.1016/j.neuron.2009.10.020 19914179

[B35] Greco S. De Simone M. Colussi C. Zaccagnini G. Fasanaro P. Pescatori M. (2009). Common micro-RNA signature in skeletal muscle damage and regeneration induced by Duchenne muscular dystrophy and acute ischemia. FASEB J. 23, 3335–3346 10.1096/fj.08-128579 19528256

[B36] Grimson A. Farh K. K. Johnston W. K. Garrett-Engele P. Lim L. P. Bartel D. P. (2007). MicroRNA targeting specificity in mammals: determinants beyond seed pairing. Mol. Cell 27, 91–105 10.1016/j.molcel.2007.06.017 17612493 PMC3800283

[B37] Hamrick M. W. Herberg S. Arounleut P. He H.-Z. Shiver A. Qi R.-Q. (2010). The adipokine leptin increases skeletal muscle mass and significantly alters skeletal muscle miRNA expression profile in aged mice. Biochem. Biophys. Res. Commun. 400, 379–383 10.1016/j.bbrc.2010.08.079 20800581 PMC3740337

[B38] Han J. Lee Y. Yeom K.-H. Kim Y.-K. Jin H. Kim V. N. (2004). The Drosha-DGCR8 complex in primary microRNA processing. Genes Dev. 18, 3016–3027 10.1101/gad.1262504 15574589 PMC535913

[B39] Han J. Lee Y. Yeom K. H. Nam J. W. Heo I. Rhee J. K. (2006). Molecular basis for the recognition of primary microRNAs by the Drosha-DGCR8 complex. Cell 125, 887–901 10.1016/j.cell.2006.03.043 16751099

[B41] Hu J. Kong M. Ye Y. Hong S. Cheng L. Jiang L. (2014b). Serum miR-206 and other muscle-specific microRNAs as non-invasive biomarkers for Duchenne muscular dystrophy. J. Neurochem. 129, 877–883 10.1111/jnc.12662 24460924

[B40] Hu Z. Klein J. D. Mitch W. E. Zhang L. Martinez I. Wang X. H. (2014a). MicroRNA-29 induces cellular senescence in aging muscle through multiple signaling pathways. Aging (Albany NY) 6, 160–175 24659628 10.18632/aging.100643 PMC4012934

[B42] Huang Z. Chen X. Yu B. He J. Chen D. (2012). MicroRNA-27a promotes myoblast proliferation by targeting myostatin. Biochem. Biophys. Res. Commun. 423, 265–269 10.1016/j.bbrc.2012.05.106 22640741

[B43] Huating W. Ramiro G. Hao S. Katherine J. L. Ravi S. Jason D. (2008). NF-κB-YY1-miR-29 regulatory circuitry in skeletal myogenesis and rhabdomyosarcoma. Cancer Cell 14, 369–381 10.1016/j.ccr.2008.10.006 18977326 PMC3829205

[B44] Hudson M. B. Woodworth-Hobbs M. E. Zheng B. Rahnert J. A. Blount M. A. Gooch J. L. (2014). miR-23a is decreased during muscle atrophy by a mechanism that includes calcineurin signaling and exosome-mediated export. Am. J. Physiol. Cell Physiol. 306, C551–C558 10.1152/ajpcell.00266.2013 24336651 PMC3948973

[B45] Hutchison E. R. Kawamoto E. M. Taub D. D. Lal A. Abdelmohsen K. Zhang Y. (2013). Evidence for miR-181 involvement in neuroinflammatory responses of astrocytes. Glia 61, 1018–1028 10.1002/glia.22483 23650073 PMC4624280

[B46] Hydbring P. Badalian-Very G. (2013). Clinical applications of microRNAs [v3; ref status: indexed, http://f1000r.es/218]. F1000Research 2:136 10.12688/f1000research.2-136.v3 24627783 PMC3917658

[B47] Jian-Fu C. Elizabeth M. M. Thomson J. M. Qiulian W. Thomas E. C. Scott M. H. (2005). The role of microRNA-1 and microRNA-133 in skeletal muscle proliferation and differentiation. Nat. Genet. 38, 228–233 10.1038/ng1725 16380711 PMC2538576

[B48] Jing X. Rongshan L. Biruh W. Yanlan D. Xiaonan W. Zhaoyong H. (2012). Transcription factor FoxO1, the dominant mediator of muscle wasting in chronic kidney disease, is inhibited by microRNA-486. Kidney Int. 82, 401–411 10.1038/ki.2012.84 22475820 PMC3393843

[B49] Kadi F. Johansson F. Johansson R. Sjöström M. Henriksson J. (2004). Effects of one bout of endurance exercise on the expression of myogenin in human quadriceps muscle. Histochem. Cell Biol. 121, 329–334 10.1007/s00418-004-0630-z 14997318

[B50] Kim H. K. Lee Y. S. Sivaprasad U. Malhotra A. Dutta A. (2006). Muscle-specific microRNA miR-206 promotes muscle differentiation. J. Cell Biol. 174, 677–687 10.1083/jcb.200603008 16923828 PMC2064311

[B51] Kuang W. Tan J. Duan Y. Duan J. Wang W. Jin F. (2009). Cyclic stretch induced miR-146a upregulation delays C2C12 myogenic differentiation through inhibition of Numb. Biochem. Biophys. Res. Commun. 378, 259–263 10.1016/j.bbrc.2008.11.041 19026611

[B52] Kukreti H. Amuthavalli K. Harikumar A. Sathiyamoorthy S. Feng P. Z. Anantharaj R. (2013). Muscle-specific MicroRNA1 (miR1) targets heat shock protein 70 (HSP70) during Dexamethasone-mediated Atrophy. J. Biol. Chem. 288, 6663–6678 10.1074/jbc.M112.390369 23297411 PMC3585106

[B53] Lecker S. H. Jagoe R. T. Gilbert A. Gomes M. Baracos V. Bailey J. (2004). Multiple types of skeletal muscle atrophy involve a common program of changes in gene expression. FASEB J. 18, 39–51 10.1096/fj.03-0610com 14718385

[B53a] Lee R. C. Feinbaum R. L. Ambros V. (1993). The *C. elegans* heterochronic gene lin-4 encodes small RNAs with antisense complementarity to lin-14. Cell 75, 843–854 10.1016/0092-8674(93)90529-Y 8252621

[B54] Lee Y. Hur I. Park S. Y. Kim Y. K. Suh M. R. Kim V. N. (2006). The role of PACT in the RNA silencing pathway. EMBO J. 25, 522–532 10.1038/sj.emboj.7600942 16424907 PMC1383527

[B55] Leung A. K. Sharp P. A. (2010). MicroRNA functions in stress responses. Mol. Cell 40, 205–215 10.1016/j.molcel.2010.09.027 20965416 PMC2996264

[B56] Lewis B. P. Burge C. B. Bartel D. P. (2005). Conserved seed pairing, often flanked by adenosines, indicates that thousands of human genes are microRNA targets. Cell 120, 15–20 10.1016/j.cell.2004.12.035 15652477

[B56a] Liang H. Ward W. F. (2006). PGC-1α: a key regulator of energy metabolism. Adv. Physiol. Educ. 30, 145–151 10.1152/advan.00052.2006 17108241

[B57] Lijun W. Liang Z. Peiyong J. Leina L. Xiaona C. Huiyao L. (2012). Loss of miR-29 in myoblasts contributes to dystrophic muscle pathogenesis. Mol. Ther. 20, 1222–1233 10.1038/mt.2012.35 22434133 PMC3369280

[B58] Lokireddy S. Wijesoma I. W. Bonala S. Wei M. Sze S. K. McFarlane C. (2012). Myostatin is a novel tumoral factor that induces cancer cachexia. Biochem. J. 446, 23–36 10.1042/BJ20112024 22621320 PMC3408049

[B59] Mayhew D. L. Kim J.-S. Cross J. M. Ferrando A. A. Bamman M. M. (2009). Translational signaling responses preceding resistance training-mediated myofiber hypertrophy in young and old humans. J. Appl. Physiol. 107, 1655–1662 10.1152/japplphysiol.91234.2008 19589955 PMC2777794

[B61] McCarthy J. J. Esser K. A. (2007). MicroRNA-1 and microRNA-133a expression are decreased during skeletal muscle hypertrophy. J. Appl. Physiol. 102, 306–313 10.1152/japplphysiol.00932.2006 17008435

[B60] McCarthy J. J. Esser K. A. Peterson C. A. Dupont-Versteegden E. E. (2009). Evidence of myomiR network regulation of β-myosin heavy chain gene expression during skeletal muscle atrophy. Physiol. Genomics 39, 219–226 10.1152/physiolgenomics.00042.2009 19690046 PMC2789671

[B62] McFarlane C. Vajjala A. Arigela H. Lokireddy S. Ge X. Bonala S. (2014). Negative auto-regulation of myostatin expression is mediated by smad3 and microRNA-27. PLoS ONE 9:e87687 10.1371/journal.pone.0087687 24498167 PMC3909192

[B63] Meister G. Tuschl T. (2004). Mechanisms of gene silencing by double-stranded RNA. Nature 431, 343–349 10.1038/nature02873 15372041

[B64] Mercken E. M. Majounie E. Ding J. Guo R. Kim J. Bernier M. (2013). Age-associated miRNA alterations in skeletal muscle from rhesus monkeys reversed by caloric restriction. Aging (Albany NY) 5, 692–703 24036467 10.18632/aging.100598 PMC3808701

[B65] Miano J. M. (2003). Serum response factor: toggling between disparate programs of gene expression. J. Mol. Cell. Cardiol. 35, 577–593 10.1016/S0022-2828(03)00110-X 12788374

[B66] Miyake T. Alli N. S. Aziz A. Knudson J. Fernando P. Megeney L. A. (2009). Cardiotrophin-1 maintains the undifferentiated state in skeletal myoblasts. J. Biol. Chem. 284, 19679–19693 10.1074/jbc.M109.017319 19439412 PMC2740593

[B67] Mizuno H. Nakamura A. Aoki Y. Ito N. Kishi S. Yamamoto K. (2011). Identification of muscle-specific micrornas in serum of muscular dystrophy animal models: promising novel blood-based markers for muscular dystrophy. PLoS ONE 6:e18388 10.1371/journal.pone.0018388 21479190 PMC3068182

[B68] Mooren F. C. Viereck J. Krüger K. Thum T. (2014). Circulating micrornas as potential biomarkers of aerobic exercise capacity. Am. J. Physiol. Heart Circ. Physiol. 306, H557–H563 10.1152/ajpheart.00711.2013 24363306 PMC3920240

[B69] Nielsen S. Åkerström T. Rinnov A. Yfanti C. Scheele C. Pedersen B. K. (2014). The miRNA plasma signature in response to acute aerobic exercise and endurance training. PLoS ONE 9:e87308 10.1371/journal.pone.0087308 24586268 PMC3929352

[B70] Nielsen S. Scheele C. Yfanti C. Åkerström T. Nielsen A. R. Pedersen B. K. (2010). Muscle specific microRNAs are regulated by endurance exercise in human skeletal muscle. J. Physiol. 588, 4029–4037 10.1113/jphysiol.2010.189860 20724368 PMC3000590

[B71] Owino V. Yang S. Y. Goldspink G. (2001). Age-related loss of skeletal muscle function and the inability to express the autocrine form of insulin-like growth factor-1 (MGF) in response to mechanical overload. FEBS Lett. 505, 259–263 10.1016/S0014-5793(01)02825-3 11566187

[B72] Panguluri S. K. Bhatnagar S. Kumar A. McCarthy J. J. Srivastava A. K. Cooper N. G. (2010). Genomic profiling of messenger RNAs and MicroRNAs reveals potential mechanisms of TWEAK-induced skeletal muscle wasting in mice. PLoS ONE 5:e8760 10.1371/journal.pone.0008760 20098732 PMC2808241

[B73] Park S.-Y. Lee J. H. Ha M. Nam J.-W. Kim V. N. (2009). miR-29 miRNAs activate p53 by targeting p85[alpha] and CDC42. Nat. Struct. Mol. Biol. 16, 23–29 10.1038/nsmb.1533 19079265

[B74] Rao P. K. Kumar R. M. Farkhondeh M. Baskerville S. Lodish H. F. (2006). Myogenic factors that regulate expression of muscle-specific microRNAs. Proc. Natl. Acad. Sci. U.S.A. 103, 8721–8726 10.1073/pnas.0602831103 16731620 PMC1482645

[B75] Roberts T. C. Coenen-Stass A. M. L. Wood M. J. A. (2014). Assessment of RT-qPCR normalization strategies for accurate quantification of extracellular microRNAs in murine serum. PLoS ONE 9:e89237 10.1371/journal.pone.0089237 24586621 PMC3929707

[B76] Russell A. P. Lamon S. Boon H. Wada S. Güller I. Brown E. L. (2013). Regulation of miRNAs in human skeletal muscle following acute endurance exercise and short-term endurance training. J. Physiol. 591, 4637–4653 10.1113/jphysiol.2013.255695 23798494 PMC3784204

[B77] Sacheck J. M. Hyatt J.-P. K. Raffaello A. Jagoe R. T. Roy R. R. Edgerton V. R. (2007). Rapid disuse and denervation atrophy involve transcriptional changes similar to those of muscle wasting during systemic diseases. FASEB J. 21, 140–155 10.1096/fj.06-6604com 17116744

[B78] Sawada S. Kon M. Wada S. Ushida T. Suzuki K. Akimoto T. (2013). Profiling of circulating MicroRNAs after a bout of acute resistance exercise in humans. PLoS ONE 8:e70823 10.1371/journal.pone.0070823 23923026 PMC3726615

[B79] Small E. M. O'Rourke J. R. Moresi V. Sutherland L. B. Mcanally J. Gerard R. D. (2010). Regulation of PI3-kinase/Akt signaling by muscle-enriched microRNA-486. Proc. Natl. Acad. Sci. U.S.A. 107, 4218–4223 10.1073/pnas.1000300107 20142475 PMC2840099

[B80] Smith S. S. Kessler C. B. Shenoy V. Rosen C. J. Delany A. M. (2012). IGF-I 3' untranslated region: strain-specific polymorphisms and motifs regulating IGF-I in osteoblasts. Endocrinology 154, 253–262 10.1210/en.2012-1476 23183171 PMC3529377

[B81] Sriram S. Subramanian S. Juvvuna P. K. Ge X. Lokireddy S. McFarlane C. D. (2014). Myostatin augments muscle-specific ring finger protein-1 expression through an NF-kB independent mechanism in SMAD3 null muscle. Mol. Endocrinol. 28, 317–330 10.1210/me.2013-1179 24438338 PMC5414928

[B82] Stepto N. K. Coffey V. G. Carey A. L. Ponnampalam A. P. Canny B. J. Powell D. (2009). Global gene expression in skeletal muscle from well-trained strength and endurance athletes. Med. Sci. Sports Exerc. 41, 546–565 10.1249/MSS.0b013e31818c6be9 19204596

[B83] Stevenson E. J. Giresi P. G. Koncarevic A. Kandarian S. C. (2003). Global analysis of gene expression patterns during disuse atrophy in rat skeletal muscle. J. Physiol. 551, 33–48 10.1113/jphysiol.2003.044701 12844509 PMC2343139

[B84] Sweetman D. Goljanek K. Rathjen T. Oustanina S. Braun T. Dalmay T. (2008). Specific requirements of MRFs for the expression of muscle specific microRNAs, miR-1, miR-206 and miR-133. Dev. Biol. 321, 491–499 10.1016/j.ydbio.2008.06.019 18619954

[B85] Thomas C. R. Blomberg K. E. M. Graham M. Samir E. L. A. Caroline G. Corinne B. (2012). Expression analysis in multiple muscle groups and serum reveals complexity in the microRNA transcriptome of the mdx mouse with implications for therapy. Mol. Ther. Nucleic Acids 1:e39 10.1038/mtna.2012.26 23344181 PMC3437806

[B86] Van Rooij E. Liu N. Olson E. N. (2008). MicroRNAs flex their muscles. Trends Genet. 24, 159–166 10.1016/j.tig.2008.01.007 18325627

[B87] Vignier N. Amor F. Fogel P. Duvallet A. Poupiot J. Charrier S. (2013). Distinctive serum miRNA profile in mouse models of striated muscular pathologies. PLoS ONE 8:e55281 10.1371/journal.pone.0055281 23418438 PMC3572119

[B88] Wada S. Kato Y. Okutsu M. Miyaki S. Suzuki K. Yan Z. (2011). Translational suppression of atrophic regulators by MicroRNA-23a integrates resistance to skeletal muscle atrophy. J. Biol. Chem. 286, 38456–38465 10.1074/jbc.M111.271270 21926429 PMC3207415

[B89] Walden T. B. Timmons J. A. Keller P. Nedergaard J. Cannon B. (2009). Distinct expression of muscle-specific MicroRNAs (myomirs) in brown adipocytes. J. Cell. Physiol. 218, 444–449 10.1002/jcp.21621 18937285

[B90] Wang L. Chen X. Zheng Y. Li F. Lu Z. Chen C. (2012a). MiR-23a inhibits myogenic differentiation through down regulation of fast myosin heavy chain isoforms. Exp. Cell Res. 318, 2324–2334 10.1016/j.yexcr.2012.06.018 22771720

[B90a] Wang L. Zhou L. Jiang P. Lu L. Chen X. Lan H. (2012b). Loss of miR-29 in myoblasts contributes to dystrophic muscle pathogenesis. Mol. Ther. 20, 1222–1233 10.1038/mt.2012.35 22434133 PMC3369280

[B91] Wei W. He H. B. Zhang W. Y. Zhang H. X. Bai J. B. Liu H. Z. (2013). miR-29 targets Akt3 to reduce proliferation and facilitate differentiation of myoblasts in skeletal muscle development. Cell Death Dis. 4:e668 10.1038/cddis.2013.184 23764849 PMC3698551

[B92] Xie W. Li Z. Li M. Xu N. Zhang Y. (2013). miR-181a and inflammation: miRNA homeostasis response to inflammatory stimuli *in vivo*. Biochem. Biophys. Res. Commun. 430, 647–652 10.1016/j.bbrc.2012.11.097 23220232

[B93] Zaharieva I. T. Calissano M. Scoto M. Preston M. Cirak S. Feng L. (2013). Dystromirs as serum biomarkers for monitoring the disease severity in duchenne muscular dystrophy. PLoS ONE 8:e80263 10.1371/journal.pone.0080263 24282529 PMC3840009

[B94] Zhao Y. Samal E. Srivastava D. (2005). Serum response factor regulates a muscle-specific microRNA that targets Hand2 during cardiogenesis. Nature 436, 214–220 10.1038/nature03817 15951802

[B95] Zhao Y. Srivastava D. (2007). A developmental view of microRNA function. Trends Biochem. Sci. 32, 189–197 10.1016/j.tibs.2007.02.006 17350266

[B96] Zhou L. Wang L. Lu L. Jiang P. Sun H. Wang H. (2012). A novel target of MicroRNA-29, Ring1 and YY1-binding Protein (Rybp), negatively regulates skeletal myogenesis. J. Biol. Chem. 287, 25255–25265 10.1074/jbc.M112.357053 22661705 PMC3408151

